# An Adaptive Kernels Layer for Deep Neural Networks Based on Spectral Analysis for Image Applications

**DOI:** 10.3390/s23031527

**Published:** 2023-01-30

**Authors:** Tariq Al Shoura, Henry Leung, Bhashyam Balaji

**Affiliations:** 1 Department of Electrical and Software Engineering, University of Calgary, 2500 University Drive NW, Calgary, AB T2N 1N4, Canada; 2 Radar Sensing and Exploitation Section, Defence Research and Development Canada, Ottawa, ON K1A 0Z4, Canada

**Keywords:** convolutional kernels, deep learning, high-resolution images, spectral analysis

## Abstract

As the pixel resolution of imaging equipment has grown larger, the images’ sizes and the number of pixels used to represent objects in images have increased accordingly, exposing an issue when dealing with larger images using the traditional deep learning models and methods, as they typically employ mechanisms such as increasing the models’ depth, which, while suitable for applications that have to be spatially invariant, such as image classification, causes issues for applications that relies on the location of the different features within the images such as object localization and change detection. This paper proposes an adaptive convolutional kernels layer (**AKL**) as an architecture that adjusts dynamically to images’ sizes in order to extract comparable spectral information from images of different sizes, improving the features’ spatial resolution without sacrificing the local receptive field (**LRF**) for various image applications, specifically those that are sensitive to objects and features locations, using the definition of Fourier transform and the relation between spectral analysis and convolution kernels. The proposed method is then tested using a Monte Carlo simulation to evaluate its performance in spectral information coverage across images of various sizes, validating its ability to maintain coverage of a ratio of the spectral domain with a variation of around 20% of the desired coverage ratio. Finally, the AKL is validated for various image applications compared to other architectures such as Inception and VGG, demonstrating its capability to match Inception v4 in image classification applications, and outperforms it as images grow larger, up to a 30% increase in accuracy in object localization for the same number of parameters.

## 1. Introduction

With the advancement of technology and the improvements of imaging equipment came a surge of very-high-resolution (**VHR**) imagery, such as the case with satellites that can now acquire images with a spatial resolution of 1 m or less, allowing the examining or features and structures in images on a closer level. However, new challenges began to emerge as “*Objects that are considered homogeneous from a semantic point of view (e.g., buildings) show a signature that is inhomogeneous at high spatial resolution because of the scattering contributions from subobjects (e.g., facade and roof in a building)*” ([1] p. 2664). Thus, re-examining the methods used to handle images is required to allow the utilization of both the previously existing features on the ones that can now be observed in larger images [2].

Convolutional neural networks (**CNNs**) have been very popular in the literature on various image applications such as image classification, object detection, and change detection, where popular models such as AlexNet [3], VGG [4], and GoogLeNet [5] became wildly used. However, as the sizes and resolutions of images increase, issues arise, requiring an in-depth look into some of the model parameters, such as the kernels’ sizes and depth needed as they need to be configured to maintain a feasible model weight to evaluate the images [6,7]. While attempts have been made to improve the models for applications on images of a larger size, most improvements focus on applications such as classification, rather than the localization, of features, where methods such as pooling and increasing the depth of the models are adopted, thus, sacrificing the spatial resolution of the features in order to increase the local receptive field (**LRF**). However, while this is convenient for image classification purposes generally, issues arise in applications such as change detection [1,2,8,9,10], because while stacking the kernels achieves the same receptive field value as the larger kernels have, this leads the deeper layers to be more biased towards pixels at the centre of the filters where weights W∼N(μ,Σ), i.e., the mean of the distribution is in the centre pixels and the least weight is towards the pixels at the corners. Furthermore, in the presence of pooling layers, while the benefit gained is the reduced dimensionality, this is achieved by throwing away information.

Another avenue of improvement can be seen in works such as the Inception layer, which is created width-wise, including different kernel sizes in order to maintain a higher LRF value while maintaining the spatial resolution of the features; however, the authors argued that “*this decision was based more on convenience rather than necessity* ([5] p. 2664)”. In later works, such as Inception v4 [11], larger kernels were added, highlighting a possible need to formulate the sizes of the kernels selected.

On the other hand, traditional implementations of image processing included using various methods such as Gabor wavelets [12] with machine learning, such as the case in [13,14,15], where the effects of selecting an appropriate filter bank in terms of size and directionality, i.e., the spectral features selection, was discussed rigorously. Furthermore, the Inception layer [5] used in GoogLeNet inherently came from [16], where the authors argued the importance of varying Gabor kernel. The authors in [5], however, opted to use trainable convolutional kernels and showed the importance of keeping the kernels features learned as it allows the model to learn features outside of the Gabor wavelets domain. Nonetheless, in addition to the fact that the kernel sizes selected were still an arbitrary choice and had to be adjusted for images of different sizes manually, an issue emerged in later versions of Inception where the kernels used were low-rank kernels, limiting the directionality of the features that can be extracted [7].

This paper proposes deploying these concepts by constructing a layer made from convolutional kernels mimicking the features that can be extracted given the Gabor wavelets rather than feeding the extracted Gabor features to a machine learning model, allowing these kernels to learn which features are of higher importance, similar to what was carried out in [5], utilizing the benefits of both Gabor features and deep learning models, where the Gabor filters allow the extraction of spectral information while maintaining the spatial resolution of the features, and deep learning models train the kernels to learn and extract better features, improving the learning as is known from their capabilities in object detection and classification. This is achieved by utilizing a known mathematical model to adaptively select which kernels to be used for different images’ sizes rather than relying on arbitrary selection, proposing the Fourier formulation as a foundation for the kernels’ architectures in terms of sizes and the number of kernels that are required in order to cover the full spectral domain using convolutional kernels, in addition to proposing a mechanism to reduce any possible redundancies and computational complexities from covering the full spectral information.

This paper is organized as follows: Section 2 provides a background review of width-based layer design in the literature, and gives the motivation behind such approaches. Section 3 examines the relation between spectral information and convolutional kernel, and establishes how convolutional kernels can be utilized to extract the full spectral information, then the section proposes an adaptive kernels layer (**AKL**) utilizing convolutional kernels and the Fourier transform definition to extract the spectral features. Section 4 presents the experiments conducted and the results obtained. Finally, Section 5 concludes this work and discusses possible research avenues in the future.

## 2. Width-Based Layer Design (Inception and Inception-like Approaches)

**Figure 1:**
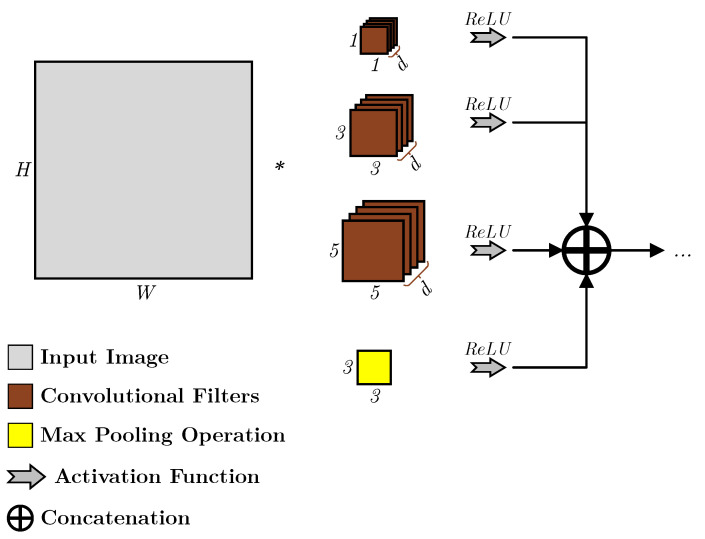
An overview of an Inception v1 layer which consists of parallel convolutional kernels and a pooling layer [5].

The Inception layer was first proposed for the ILSVRC-2014 competition, and since then, it has progressed through different iterations and become one of the cornerstone models for image processing using deep learning. The Inception tries to solve a problem that is caused by the fact that the size and location of the information in the image varies immensely by introducing what is now known as the “naive” Inception layer, or Inception v1, where filter sizes of 1×1, 3×3, and 5×5 in addition to a max pooling layer were chosen for the implementation, as shown in Figure 1, where the outputs are then concatenated and sent to the next layer. However, another issue emerged as the sizes of images increased, where the features’ sizes in the images were also scaled, requiring the need for larger kernels.

**Figure 2:**
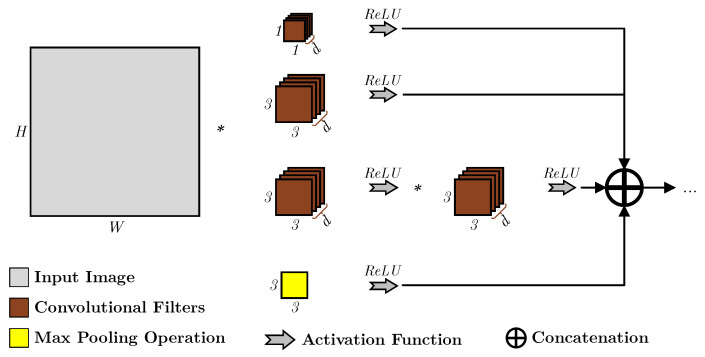
An overview of an Inception v2 layer which replaced the 5×5 with two stacked 3×3 convolutional kernels [17].

The next iterations of Inception—namely, Inception v2 and Inception v3—realized this issue [17]; thus, the kernels of LRF value of 7×7 were included in the model. However, as the focus was on attempting to increase the speed of the model’s computations, the authors opted to break down the convolutional operations, where 5×5 convolution was replaced by two stacked 3×3 convolutional operations, as shown in Figure 2, boosting the speed of the layer, although it sacrifices the spatial resolution of the features.

**Figure 3:**
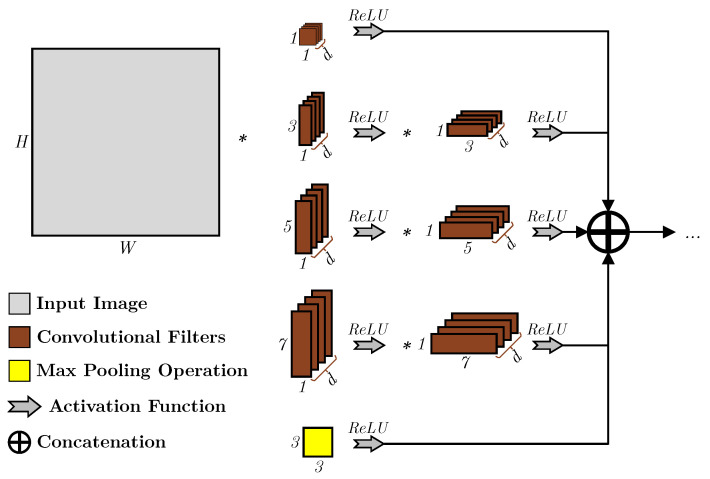
An example of an Inception v4 layer that employs larger kernel sizes and utilizes low-rank kernels for the implementation [11].

Furthermore, convolution factorization was also introduced and heavily used in the next iterations, such as the Inception v4 layer [11], shown in Figure 3, to improve the speed further, where low-rank kernels are used for the construction of the convolutional kernels by breaking down the traditional convolutional kernel matrix of size *K* into two vectors, *u* and *v*, such that u×1×v=K, where the aim of this is to reduce the number of computational operations required as the kernels size increase. However, only a minority of kernels are spatially separable, which limits the number of features that can be learned when utilizing the larger kernels.

**Figure 4:**
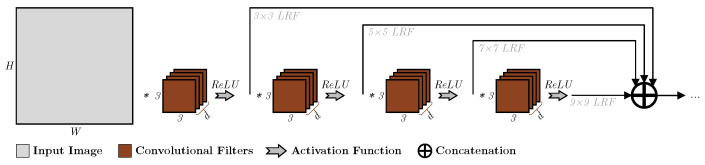
An example of an Inception-like layer that uses deep convolution and residual connections to emulate the behaviour of an Inception layer in utilizing multiple LRFs at the output.

**Figure 5:**
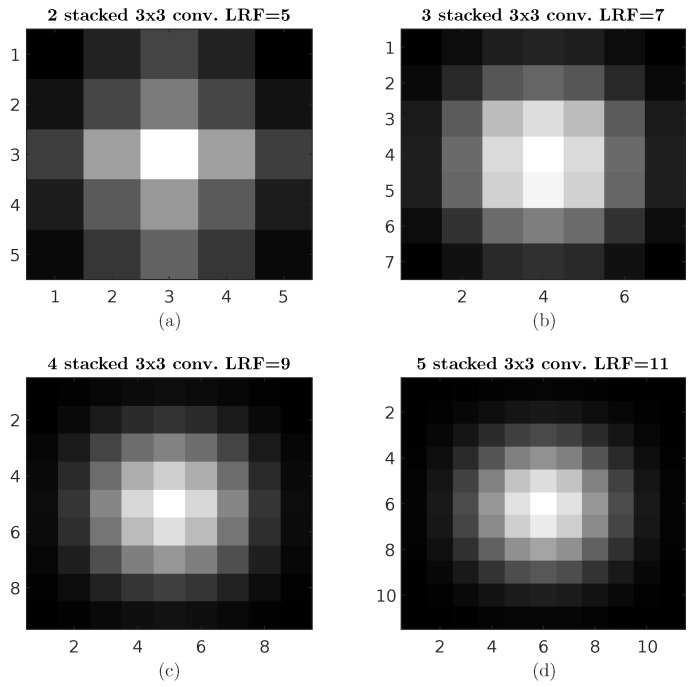
Stacking convolutional kernels’ effect on spatial resolution, where it can be seen that as the number of stacked layers increases, the output results favour the results toward the centre more, where the lowest weights are towards the corners of the input.

Other works, such as [18,19,20,21,22,23,24,25], implemented what is known as Inception-like blocks, which use deep conventional networks, and the residual connections introduced in [26] to extract the outputs from each layer and concatenated them for the output, as shown in the example in Figure 4, mimicking the operation using the Inception layer depth-wise by allowing the extracted feature at multiple receptive fields to be processed at the output layer. However, while this approach can be suitable for certain applications such as classification, a drop in the spatial features accumulates as we move deeper, diminishing the spatial accuracy of the larger LRF values, as illustrated in Figure 5, where it can be seen that a bias towards features at the centre starts to increase, impairing the capability of the layer to accurately position where the feature is located and decreasing its efficiency in applications such as object detection.

## 3. Proposed Method: An Adaptive Kernels Layer

### 3.1. Examining Spatial–Spectral Features and Convolutional Kernels

As the resolution and size of images change, objects that appear in images are now represented by a different number of pixels. However, when examined through spectral analysis, it can be seen that the same objects remain represented at similar frequencies relative to the images’ size; this is because while the number of pixels used to represent an object changed, the ratio between the number of pixels representing an object and the total amount of pixels in the image is still preserved, regardless of the resolution of the images. Thus, the feature of interest that is focused on in this paper is the rate at which the pixel intensities around a pixel *P* change given a local neighbourhood of size *N*, where *N* is considered at different values reflecting the different possible images of resolutions.

The concept of examining the rates of change is not a foreign concept to the literature of image processing, as methods that allow examining multiple rates of change at once exist, where one of the most known examples to examine the rate of change of observations in the data is the Fourier transform, as it extracts the different frequencies of a signal, where the frequency is the number or the rate of wavelets that pass given a set amount of time, i.e., the number or rate of changes between peaks and valleys within a given period. However, when dealing with images, the rate of change here is the rate at which the pixels’ illumination values change. Furthermore, since images are two-dimensional, the two-dimensional Fourier transform can be used to study the rate of change which is now made of two components: the magnitude and the direction of the change, where the amount of change observed as part of the spectral transform can be seen in the overall amplitude at any given frequency and direction.

Once the full spectral information of an image is obtained, bandpass filters can be utilized to extract specific spectral features’ components to be examined separately, and careful selection of those frequencies is imperative given that different frequencies represent different features of objects; for example, low frequencies typically represent the overall illumination of objects and slower changes across the image such as large buildings or a face in a portrait, and, on the other hand, higher frequencies are more capable of distinguishing changes in textures and boundaries of objects such as edges in certain directions, or the textures of the objects. The importance of this feature selection can be seen in the literature for various applications such as edge and face detection.

**Figure 6:**
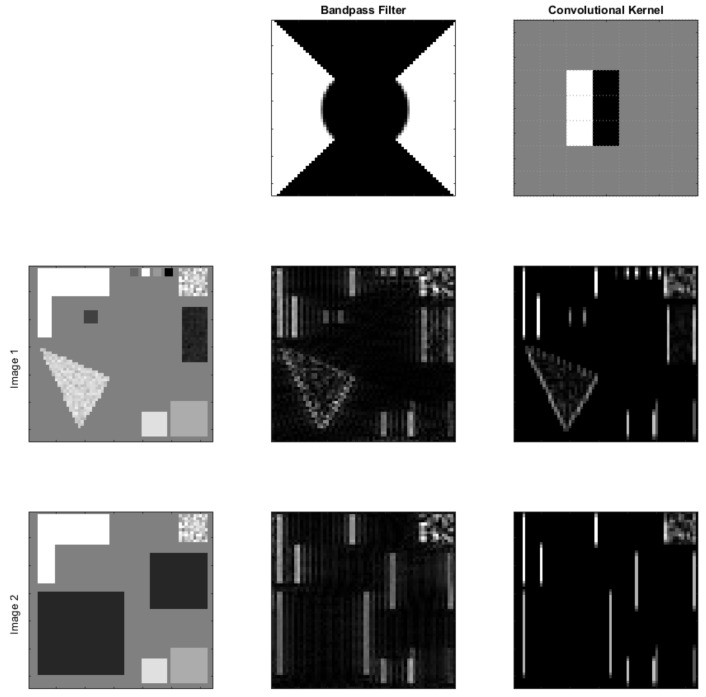
An example of how the features of a bandpass filter can be extracted using convolutional kernels, where the figure shows vertical image detection using a bandpass filter and convolutional kernel on two sample images.

A popular example of bandpass filters is the Gabor transform, where a Gabor wavelet is convolved with the input image such that the output is a map indicating where the features of the image exist in the image itself, maintaining the spatial resolution of the features, unlike Fourier transform. As was discussed earlier, the motivation for Inception stems from the need for various Gabor wavelets, and given that traditional convolutional filters can be used to extract features similar to those that can be extracted using bandpass filters, as can be seen in Figure 6, in addition to the fact that convolutional kernels have the ability to learn features that can be most useful for deep learning models, a question presents itself: How can convolutional kernels’ architecture be designed to represent the features of different frequencies and orientations?

### 3.2. Proposed Layer Formulation

For any feature that spans over a given number of pixels *k*, the maximum number of times that this feature can occur in an image *I* of size *N* is k/N, and while this feature can occur a fewer number of times within the image, this at least indicates that for any given rate of change, the size of the kernel needed to represent that feature is ≥k. Furthermore, since it was established when examining Fourier transform that the features, i.e., the rate of changes, is a function of both the magnitude and the direction of the change, utilizing different sizes of kernels covers the requirement for the magnitude, and the arrangement of the values within those kernels covers the directional requirement.

The first decision made was to build the layer width-wise to be used directly after the input; this was performed in order to maintain the highest possible spatial resolution of the extracted features, given that the local receptive field value at the input layer is the same as the kernels’ size. The next step is determining what kernels are needed to provide the next layers with features; thus, the definition of the Fourier transform formulation is used to maximize the spectral features coverage.

The proposed solution utilizes multiple convolutional kernels of different sizes at the input level, as each convolutional kernel will be utilized in a matter similar to a bandpass for the given filter size such that $C_{k}$ is the convolutional kernel that detects the feature $f_{k}$, where $C_{k}\left(I\right)$ indicates the response of a convolutional kernel of size *k* when applied to image *I*. However, an issue remains where the number of discrete constructed orientations for features of size has to be determined in order to generate an adequate number of kernels to cover the directionality.

A simple way to examine this can be by identifying the number of fractions that can be created, using any number *k* as a denominator which equals (k−1). However, these fractions only represent half of the first quadrant’s possible ratios; thus, in order to cover the first two quadrants, the number of kernels of size *k* that are needed should be at least 4×(k−1). This leads to a set of concatenated kernels $C_{k}=\left\{C_{k_{1}}\left|\right|C_{k_{2}}\left||\cdots |\right|C_{k_{4k-4}}\right\}$, where || denotes the concatenation operation, and by performing this for every possible feature size *k* that exists within the image of size *N*, we generate a superset of kernels $K=\left\{C_{2}\left|\right|C_{3}\left||\cdots |\right|C_{N-1}\right\}$ which approximates the Fourier transform.

However, while performing this covers the whole spectrum, it includes some mathematical redundancy as a feature $f_{a}$ can be represented using a feature $f_{b}$ given that a≤b, which means that larger kernel can inherently include smaller kernels, meaning that it is possible to remove some of the smaller kernels from the set if the number of the larger kernels increases. However, assuming possible kernel cell values in a set *V*, the number of possible kernels of size *k* is given by $size{\left(V\right)}^{k^{2}}$; this means that $P\left(C_{b}\in C_{a}\right)\propto 1/\left(b^{2}-a^{2}\right)$, meaning that selecting only larger kernels can be problematic as it statistically leads to the loss of smaller features, i.e., the high-frequency components.

To accommodate for this issue, this paper proposes using kernel sizes that correspond to the set of prime numbers P; this is because the prime numbers have adequate spacing between them, in addition to the fact that all the possible fractions in an image sample space can be covered by them since any i/P is inherently a unique fraction, where *i* is any positive nonzero integer less than *P*.

Furthermore, to allow the layer to be adaptive to the size of the images *N*, the kernel sizes *k* are made of an adaptive set such that k∈3≤P≤N/w, where *w* is a control variable that determines the lowest frequency to be considered, where, experimentally, the value of w=4 was found to be adequate generally, covering approximately 25% of the overall existing frequencies; however, depending on the application, this value can be changed.

Additionally, due to the unique capabilities of the convolutional layer to extract features other than the ones that are part of the Fourier space, a control variable *c* is introduced, allowing the number of kernels to be increased, where it was experimentally found that at least doubling the number of kernels for change detection applications by setting the value of c=2 allows these features to be extracted, in addition to allowing the existing kernels to cover the features of sizes that were excluded, too.

**Figure 7:**
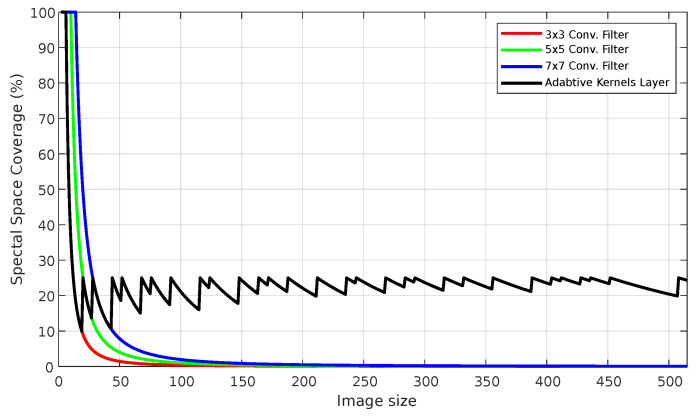
AKL vs. fixed kernel size coverage of spectral space as images’ sizes increase.

To better examine this, a Monte Carlo simulation across various image sizes was performed to measure the AKL’s ability to cover the spectral information, shown in Figure 7, where it can be seen that with w=4, the ratio is maintained between 20–25% as images grow larger; this is because the definition implicitly allows for kernels of new sizes to be added, allowing more features to be included.

**Figure 8:**
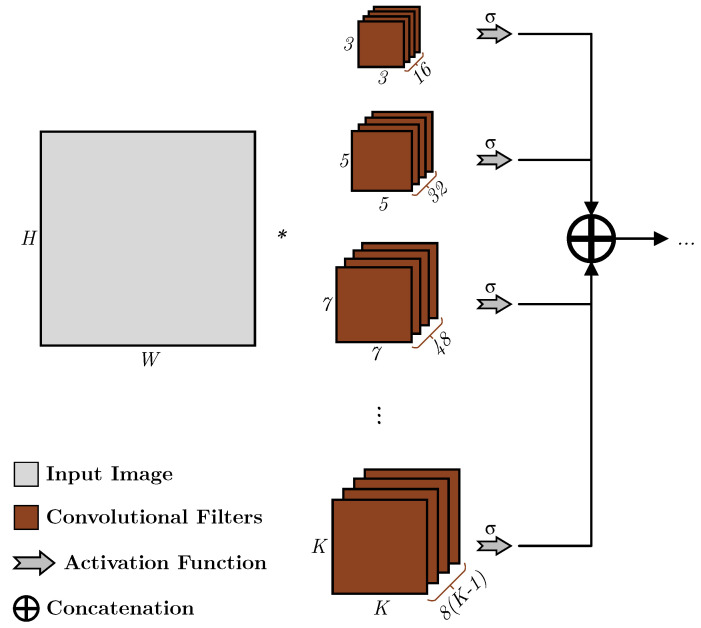
An overview of the adaptive kernels layer using c=2.

The outputs of the layers of different sizes are concatenated without any pooling to maintain the spatial resolution of the information obtained; thus, the AKL can be represented as shown in Equation (1), where $K_{k}^{i}$ indicates $i^{th}$ kernel of size *k*, *I* is an image of size $i_{1}\times i_{2}$, $C_{k_{i}}\left(I\right)$ is the convolutional response of image *I* using the kernel $K_{k}^{i}$, *w* and *c* are parameters that are introduced to allow limiting the spectral coverage required and the number of kernels generated, respectively, and *N* is the size of the image. Figure 8 illustrates the layout of the proposed architecture.

(1) $$\begin{array}{cc} AKL(I)= & \;\;K*I\\ = & \;\;C_{3_{1}}\left(I\right)||C_{3_{2}}\left(I\right)||\cdots ||C_{k_{i}}\left(I\right);\\ \; & \;\;\;\;\;\;\;\;\forall k\in \{3\leq \left(P\right)\leq \frac{N}{w}\},\forall i\leq c\left(4k-4\right) \end{array}$$

where

$$\begin{array}{cc} C_{k_{i}}\left(I\right)=\sum_{a=1}^{k}\sum_{b=1}^{k}K_{k}^{i}\left(a,b\right)\cdot I( & u+a,v+b);\\ & \forall u\in i_{1},\forall v\in i_{2} \end{array}$$

**Figure 9:**
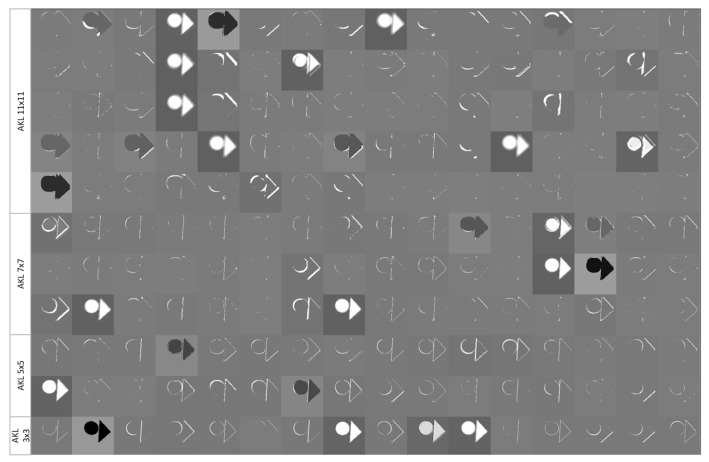
Sample output of the features learned by AKL’s kernels.

Another concern when implementing the AKL stems from the fact that typical implementations of the neural network utilize libraries such as TensorFlow for rapid development and convenience. When applying AKL, one issue that arises is from the fact that the different convolutional kernel sizes will output results of various sizes if they are applied to the confines of the images themselves. In order to accommodate for that, the images are padded on the edges for a given kernel size *k* with $\frac{k-1}{2}$ pixels; these can either be static zero pixels, but preferably a copy of the nearest true pixel value, or an average of the surrounding pixels. While the padding could be of concern as it dilutes the data itself, the smaller kernel sizes, in addition to the aggregation of the kernels, would help in consolidating the data on the image’s edges. Furthermore, larger kernels are utilized to extract the low-frequency features within the images; hence, their response on the edges of an image is of lower importance. Figure 9 shows a sample output of the feature learned at the different levels of AKL, where it can be seen that the larger kernels are able to isolate finer parts of the objects without the need to rely on increasing the depth and sacrificing the accuracy of the features’ location.

Finally, the selected kernels are implemented as k×k kernel directly rather than using low-rank kernels; this is because, assuming that *K* is a k×k matrix, the construction of any given *K* using two vectors *u*, *v* such that K=u×v dictates that there exists $v=u^{-1}K$, and since *u* is a vector, the inverse can be obtained using the pseudo-inverse denoted as $u^{+}$. However, the conditions for a solution to exist are defined by whether $AA^{+}b=b$ given Ax=b, and this condition does not hold when the sizes of the vectors are considered.

## 4. Experimental Results

This section tests the validity of AKL compared to other feature extraction architectures such as Inception and VGG. The models were tested in various image-related applications such as classification and object detection for datasets of various image sizes. This section presents and discusses the experimental results obtained for the various applications. For this section, the parameters used for AKL were w=4 and c=2 as they allowed for a comparable number of parameters (5.86–31.7 M) of largest kernel size against Inception v4 (5.86–34.6 M) to allow for a fair comparison.

### 4.1. Image Classification

To test the validity of the features extracted by the AKL, an experiment using different datasets was conducted to test the capability of feature learning for images of various sizes, which is performed by utilizing the datasets below, given their different image sizes:

- Horses or Humans Dataset [27]: consists of about 1000 rendered images of size 300×300, divided into two categories: horse or human.
- Cats and Dogs Dataset [28]: this dataset was generated for the Playground Prediction Competition, and is made from 25,000 images of sizes 500×500, divided into two categories: cat or dog.
- Food 5K Dataset [29]: consists of 50,000 images of size 1024×735 divided into two categories: food or nonfood.

In order to test the different CNN architectures’ features’ impact on image classification applications, multiple deep learning models were created, where the input layer was changed for each of the CNN architectures, and then the models were trained for the different datasets. All the outputs for the different models were kept the same in order to ensure that the evaluation was only based on the impact of the input features learned, where the features were then fed into a dense layer with a number of neurons equivalent to the number of classes. All the different models were trained for the same number of iterations to establish a balanced comparison, where it should be mentioned that given a dense network for the output, all models were able to achieve a prediction accuracy larger than 99%, given an 80−−20 split of the data between training and testing. Finally, the output neurons were activated as softmax and the models were trained using categorical cross-entropy.

The tests were conducted using Python 3.7.7 and TensorFlow 2.1.0 on a machine with an Intel i7-8750H CPU and an NVIDIA GTX 1070 GPU with 8 GB of VRAM. We used the Adam optimizer [30] with a learning rate = 0.01, and decay = $10^{-7}$, where the models were trained for 50 epochs, keeping the best-trained model with a batch size of the changesbased on the dataset’s images’ sizes.

**Figure 10:**
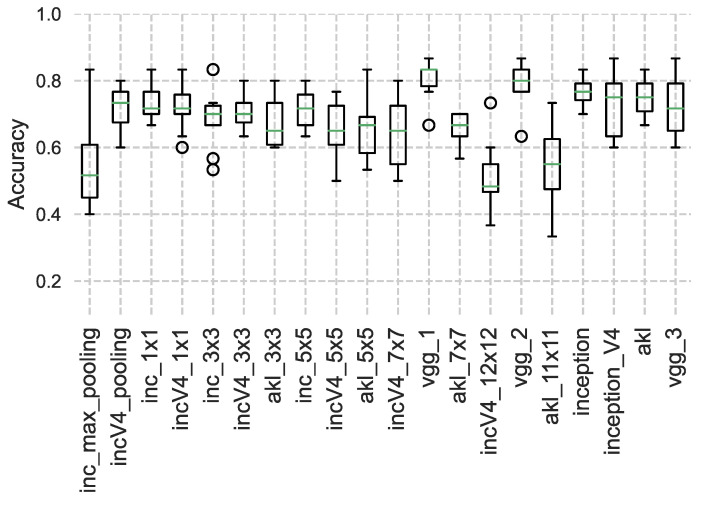
K-NN results using the Horse vs. Human dataset. The performance of the larger kernels, i.e., incV4_12×12 and akl_11×11, is low compared to the other kernels for the images of smaller size.

**Figure 11:**
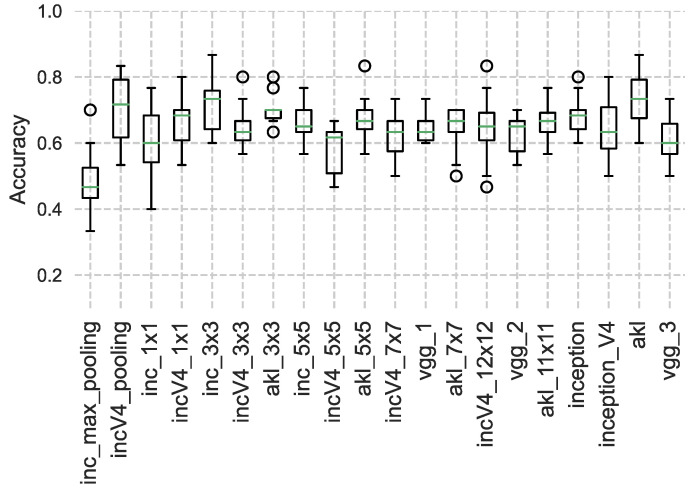
K-NN results using the Cat vs. Dog dataset. The results of all kernels are comparable as the images are of relatively medium size.

**Figure 12:**
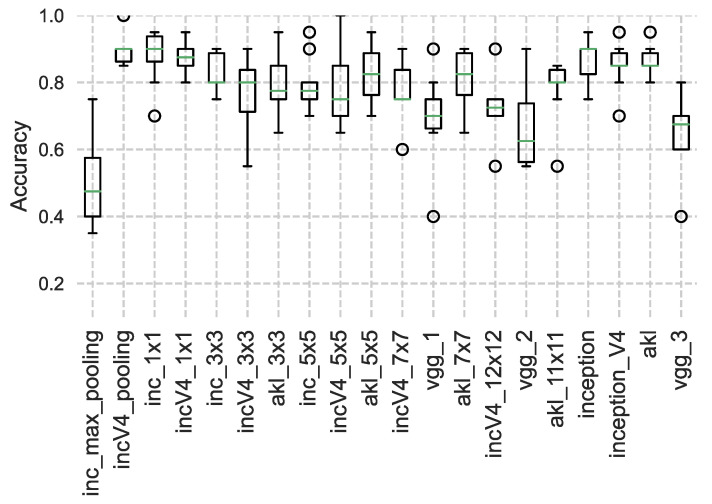
K-NN results using the Food-5K dataset. A performance drop in the deeper VGG layers can be seen as the size of the images is increased.

Once a model has been trained, the output features of the different architectures are fed into a K-nearest neighbour (**K-NN**) [31] classifier as another method to examine the extracted features’ performance and overall generalization, rather than just using dense networks, where Figure 10, Figure 11 and Figure 12 show the accuracy results obtained using the clustering method through the different datasets and extracted features, where the accuracy is given as the ratio of the number of the correct predictions over the total number of samples. This step was performed in an attempt to try to evaluate the robustness of the features learned from each kernel size and method, where the quality of the learned features is assessed through how well a classification algorithm performs using the features learned by the kernel only.

The procedure of generating a new instance of each layer’s architecture, training the filters using a dense network, feeding the outputs to K-NN, and evaluating the predictions was then repeated 50 times to have an adequate sample to be able to compare the different architectures. The same tests were conducted again on a different machine to validate the results using Python 3.8.3 and TensorFlow 2.1.0, running on an Intel Xeon E5-2699A and a Tesla V100-SXM2-16 GB GPU with 16 GB of VRAM, using the same optimizer and configurations.

It can be seen from the results that for datasets with smaller images, the features extracted from the larger kernels of Inception and AKL were not able to produce good results, unlike the case of VGG’s blocks, which, even though they share the same local receptive field values, the features that are learned at that depth are established given the weights of earlier layers in the network, improving their performance. However, as the images’ sizes increase, the larger kernels start to produce better results when used with the classifier, where it can be seen that the overall performance of VGG drops compared to the Inception and AKL layers, since, unlike VGG, which provides a single local receptive field for the classifier, these methods provide more insight into the features contained within the images for the classifier to use. Finally, the effects of the low-rank kernels start to appear when comparing the larger kernels of Inception v4 with their counterparts from AKL, where, as was discussed, while utilizing the low-rank kernels reduces the computation, it affects the range of features that can be detected. However, while this may affect the performance of the individual kernels, the aggregate of kernels’ sizes still provides robust features to be used for classification.

### 4.2. Object Localization

While examining the effect of the kernels for classification application can provide an insight into the quality of the features, another point that has to be considered when evaluating the extracted features is their spatial resolution, i.e., the relation between the extracted features and their actual location in the image. However, while there is no standard defined way to measure that, studying how well the different architectures perform in applications such as object localization can help establish an understanding.

To evaluate the object localization, a random object in terms of shape and size is placed into an image at a random location and a random size, where the models are trained given three parameters that define the bounding box around the object, which are the x-location, the y-location, and the size of the box, representing the three required outputs which are activated using sigmoid, where the models were trained using binary cross-entropy.

**Figure 13:**
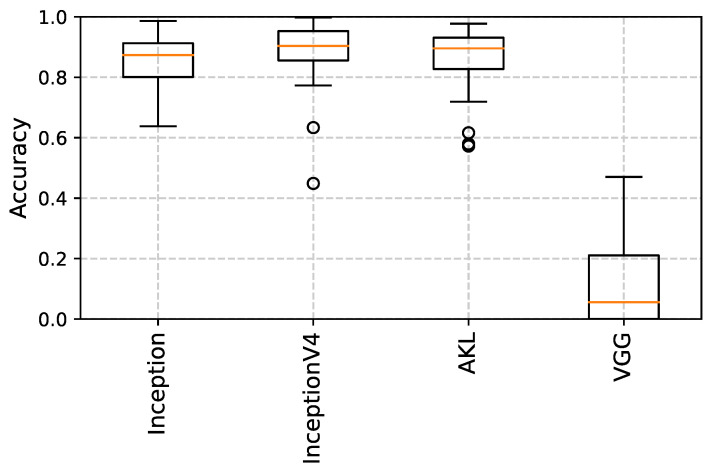
IoU of object localization of different CNN architectures for images of size 128×128. The performance of the width-based architectures is better than the stacking-based architectures.

**Figure 14:**
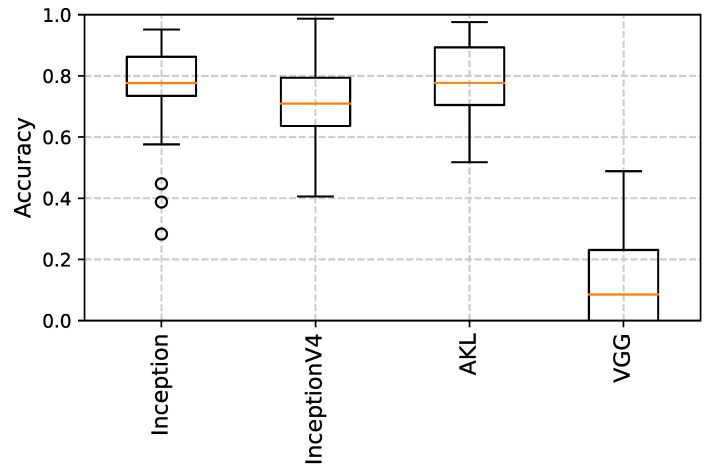
IoU of object localization of different CNN architectures for images of size 256×256.

**Figure 15:**
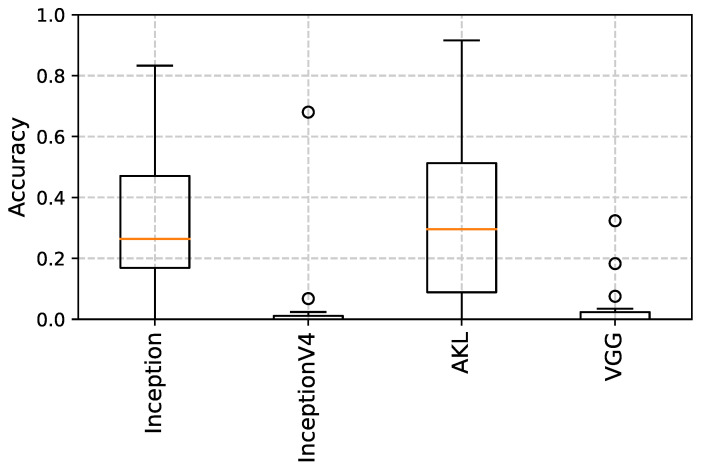
IoU of object localization of different CNN architectures for images of size 512×512. There is a noticeable drop in performance of Inception_v4.

Multiple models were created where only the first layer was changed to each of the CNN architectures. The models were first trained to detect an object that appears in an image for 30 epochs, where the objects are generated following a uniform random distribution in terms of location and size, which are the expected outputs of the trained models. The models are then tested using another random set of objects, where the outputs are evaluated using the intersection over the union (**IoU**) [32] between the true bounding box and the predicted bounding box. The test was conducted on images of different sizes from 128×128 to 512×512, where the training set used 1024 random objects and the testing was performed over 256 random objects, where the results can be seen in Figure 13, Figure 14 and Figure 15.

The tests were conducted using Python 3.7.7 and TensorFlow 2.1.0 on a machine with an Intel i7-8750H CPU and an NVIDIA GTX 1070 GPU with 8 GB of VRAM. We used the Adam optimizer with a learning rate = 0.01 and decay = $10^{-7}$, where the models were trained for 50 epochs, keeping the best-trained model with a batch size of the changesbased on the dataset’s images’ sizes. The results were then validated by performing the same tests on a different machine using Python 3.8.3 and TensorFlow 2.1.0, running on an Intel Xeon E5-2699A and a Tesla V100-SXM2-16 GB GPU with 16 GB of VRAM, using the same optimizer and configurations.

From the results, it can be seen that VGG, a stacking-based architecture, produced the worst results when it came to object localization, which matches the expected outcome due to the loss of spatial resolution due to the stacking operation. Moreover, it can be seen that while Inception v4 performed well for smaller images, a quick drop in performance occurred as images become larger, and while the cause of this needs a deeper examination, it can be inferred from the fact that Inception v1 and AKL maintained a slightly better performance that this could be due to the use of the low-rank filters in Inception v4. Finally, it should be noted that the AKL filters’ sizes were capped at 7 × 7 to insure a fairer evaluation against the Inception architectures.

## 5. Conclusions and Future Work

This paper shows the potential of re-examining convolutional kernels design width-wise and approaches this from a spectral analysis point of view by proposing an initial formulation based on the Fourier transform definition for the selection of the kernel, especially when it comes to dealing with larger images, where it can be seen that it produces comparable results to the Inception v4 in image classification applications, and at least matches or shows improvements of up to 30% in performance for some cases in applications such as object localization for larger images. While its effectiveness on larger images can be seen from the experiments conducted, there is still a large amount of room for potential experiments and improvements to the design to allow the layer to be useful even in applications with smaller images, where examples of possible improvements include a deeper examination into the AKL’s parameters and how they affect different image applications, examining the ratio of the number of kernels used for each kernel size, initializing the kernels in other configurations such as Gabor wavelets, and the utilization of other definitions that can be used to select the kernel sizes to be utilized.

Other avenues of research include the utilization of AKL in other fields such as image segmentation, change detection, image registration, etc., in addition to studies on different types of images, such as MRI images and remote sensing images, to examine the effect of the proposed layer. Moreover, other avenues of research that can be considered are how AKL interacts with and affect other deep learning components such as RNNs, residual networks (**ResNets**), and attention mechanisms.

## Data Availability

Not applicable.

## Abbreviations

The following abbreviations are used in this manuscript:

VHR	Very high resolution
CNN	Convolutional neural networks
LRF	Local receptive field
AKL	Adaptive kernels layer
IoU	Intersection over the union
